# Rethinking small vessel CTOs: collateral channels as a bridge to subsequent complex intervention-a case report

**DOI:** 10.1093/ehjcr/ytag241

**Published:** 2026-04-21

**Authors:** Zizhuo Su, Jiajie Li, Woliang Yuan, Jingfeng Wang, Maohuan Lin

**Affiliations:** Department of Cardiology, Sun Yat-Sen Memorial Hospital, Sun Yat-Sen University, Guangzhou 510120, China; Guangdong Province Key Laboratory of Arrhythmia and Electrophysiology, Guangzhou 510120, China; Department of Cardiology, Sun Yat-Sen Memorial Hospital, Sun Yat-Sen University, Guangzhou 510120, China; Guangdong Province Key Laboratory of Arrhythmia and Electrophysiology, Guangzhou 510120, China; Department of Cardiology, Sun Yat-Sen Memorial Hospital, Sun Yat-Sen University, Guangzhou 510120, China; Guangdong Province Key Laboratory of Arrhythmia and Electrophysiology, Guangzhou 510120, China; Department of Cardiology, Sun Yat-Sen Memorial Hospital, Sun Yat-Sen University, Guangzhou 510120, China; Guangdong Province Key Laboratory of Arrhythmia and Electrophysiology, Guangzhou 510120, China; Department of Cardiology, Sun Yat-Sen Memorial Hospital, Sun Yat-Sen University, Guangzhou 510120, China; Guangdong Province Key Laboratory of Arrhythmia and Electrophysiology, Guangzhou 510120, China

**Keywords:** Chronic total occlusion, Collateral channel, Coronary small vessel, Retrograde approach, Percutaneous coronary intervention, Case report

## Abstract

**Background:**

The retrograde approach has been integrated into global practice for managing challenging chronic total occlusion (CTO). A fundamental limitation of this technique is the requisite presence of navigable collateral channels. Thus, the incomplete exposure of collateral pathways renders complete interventional revascularisation of multi-vessel CTOs a persistent challenge.

**Case summary:**

A 53-year-old man was admitted for unstable angina. Coronary angiography demonstrated three-vessel CTOs, with a long right coronary artery (RCA) CTO, a proximal left anterior descending artery (LAD) CTO, and a CTO in a small-calibre left circumflex (LCX). A staged percutaneous coronary intervention (PCI) approach was adopted. The LAD and LCX CTOs were sequentially revascularized via an antegrade approach. Leveraging optimal visualisation of the collateral channels, particularly an interventional channel from the distal LCX to the RCA, a more challenging RCA CTO was successfully revascularized using a retrograde approach. The patient was symptom-free and was discharged 3 days later.

**Discussion:**

This case highlights the presence of potential interventional collateral channels in a small vessel CTO, which may enhance the success rate of subsequent, more complex CTO interventions via a retrograde approach.

Learning points

- Meticulously reviewing the angiogram is mandatory when planning a staged PCI strategy for multi-vessel CTOs.
- The potential collateral channels exposed in small vessel CTOs may provide retrograde access to facilitate revascularisation of subsequent, more complex CTOs.

## Introduction

Sole reliance on the antegrade approach is frequently insufficient to ensure technical success in highly complex chronic total occlusion (CTO) interventions. The introduction of the retrograde approach has markedly increased the success rate for such challenging lesions,^1^ a technique that hinges on the presence of suitable interventional collateral channels.^2,3^ A key persisting hurdle in multi-vessel CTOs is the difficulty of achieving complete percutaneous revascularisation, often attributable to incomplete delineation of collateral pathways. Notably, the sequential treatment of one CTO lesion can unmask collaterals supplying another, thereby potentially enhancing the success of subsequent interventions. The prognostic benefit of revascularising the small coronary vessels remains controversial.^4–7^ Consequently, percutaneous coronary intervention (PCI) for CTOs located in small vessels is rarely indicated in isolation. Nevertheless, this case underscores a strategic rationale for small-vessel CTOs PCI within multivessel disease: the collateral networks it reveals can provide potential retrograde access to facilitate the treatment of subsequent, more complex CTOs.

## Summary figure

**Figure:**
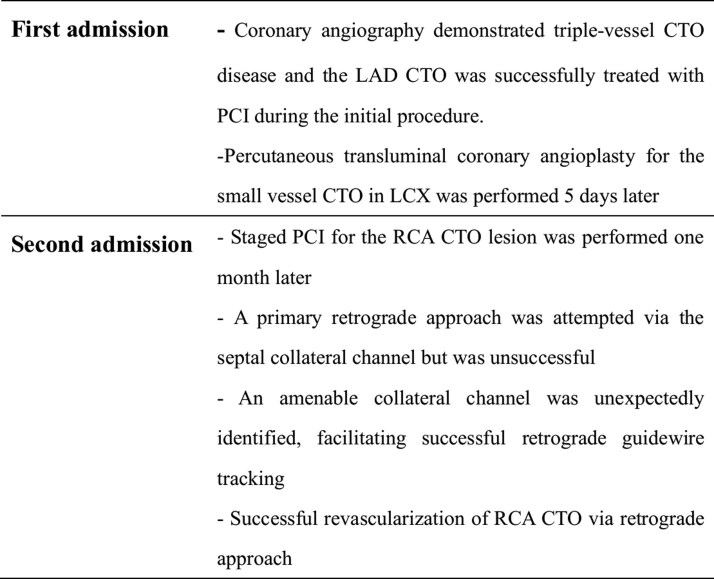


## Case presentation

**Figure 1:**
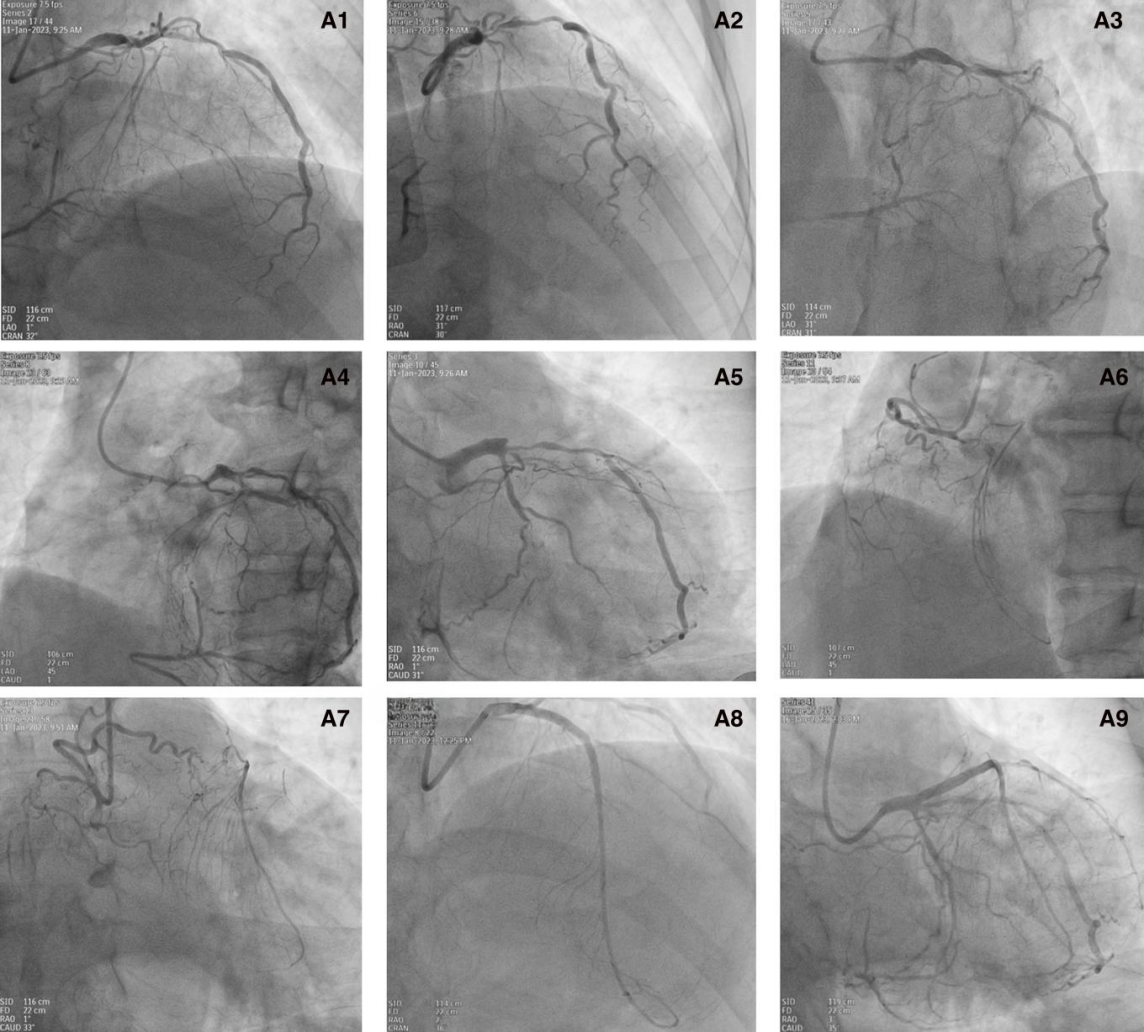
Sequential revascularisation of LAD and LCX CTOs. A1-A7) Baseline angiographic anatomy of the left and right coronary arteries. A8) Final result following percutaneous coronary intervention (PCI) of the left anterior descending (LAD) artery. A9) Final result following percutaneous transluminal coronary angioplasty (PTCA) of the left circumflex (LCX) artery.

A 53-year-old man with hypertension was admitted for unstable angina. Coronary angiography revealed three CTOs located in the proximal left anterior descending artery (LAD), the distal left circumflex artery (LCX), and the proximal right coronary artery (RCA) (*Figure 1 A1 to A7* and Moving image  *S1*  to  *S7*). The patient declined coronary artery bypass grafting (CABG). Therefore, a strategy of complete revascularisation with staged PCI was undertaken. The initial target was the LAD CTO, which was recanalized via antegrade wire escalation (AWE) and treated with three drug-eluting stents (2.5 × 18 mm, 3.0 × 18 mm and 3.5 × 18 mm, *Figure 1 A8* and Moving image  *S8*).

Five days later, the small vessel CTO in the LCX was subsequently addressed with percutaneous transluminal coronary angioplasty (PTCA) using a drug-coated balloon (*Figure 1 A9* and Moving image  *S9*).

**Figure 2:**
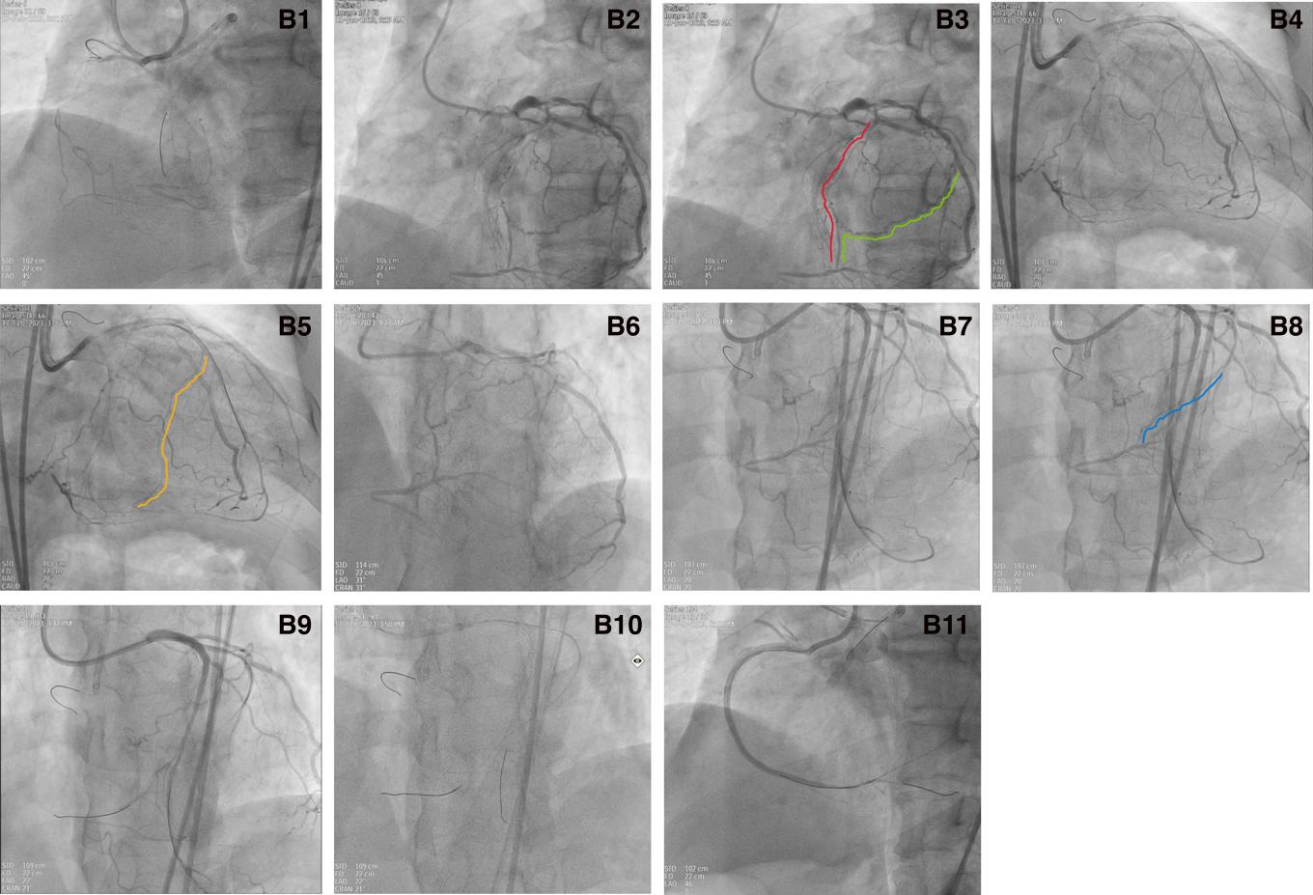
Procedural steps of RCA CTO recanalisation via the retrograde approach. B1) Dual injection confirming bilateral filling. B2, B3) Angiograms demonstrate collateral channel 1 (red) and channel 2 (green) from the AV groove to the PLV. B4, B5) Angiogram demonstrating collateral channel 3 (orange) from the septal to the posterior descending branch. B6) Pre-intervention angiogram revealing the absence of a favourable channel before LCX PTCA. B7, B8) Post-LCX PTCA angiograms revealing the newly exposed and ideal collateral channel 4 (blue) from the AV groove to the PLV. B9) The Sion guidewire successfully navigated channel 4 to the distal cap of the RCA CTO. B10) The Caravel microcatheter advanced retrograde into the distal RCA. B11) Final angiographic results after successful RCA CTO stenting.

The RCA CTO presented as a long, multi-segmental occlusion with two notable features: the lack of a proximal tapered stump and a distal cap terminating at a bifurcation. Given that the RCA CTO's anatomy—a long occlusion without a proximal stump and a bifurcated distal cap—predicted a low success rate with antegrade techniques and was unsuitable for antegrade dissection and re-entry (ADR), a retrograde approach was therefore pursued to optimize the likelihood of procedural success. Baseline coronary angiography demonstrated at least two collateral channels, comprising two atrioventricular (AV) groove collateral channels (channel 1 and channel 2, J-channel score of both channels ≥2, *Figure 2 B2* and *B3*, and Moving image  *S5*). Moreover, revascularisation of both the LAD and the LCX may expose additional septal or epicardial collateral channels by altering collateral flow dynamics, thereby creating new potential retrograde access routes. One month later, the patient underwent PCI for the RCA CTO. Following dual injection (*Figure 2 B1*, Moving image  *S10*  to  *S12*), we interrogated for collateral channels from the LAD to the RCA and identified a septal collateral channel connecting to the posterior descending branch (channel 3, J-channel score 2, *Figure 2 B4* and *B5*, and Moving image  *S11*). A primary retrograde strategy was employed. The initial attempt was made to cross the septal collateral channel 3. However, a series of guidewires (Sion, Sion Black, Suoh 03) were attempted, but all were unsuccessful in crossing the septal collateral channel to the posterior descending branch. Prior to attempting collateral channels 2 or 3, the angiograms were re-evaluated. Upon re-evaluation, we serendipitously identified a previously non-viable and more favourable AV groove collateral channel, originating from a distal branch of the LCX and connecting to the posterior left ventricular (PLV) branches (channel 4, J-channel score 2, *Figure 2 B6 to B8*, Moving image  *S3* and *S12*). We successfully negotiated collateral channel 4 with a Sion guidewire and a Caravel microcatheter, advancing them sequentially to the PLV branch (*Figure 2 B9* and *B10*, Moving image  *S13* and *S14*). Following the creation of a pathway using the reverse controlled antegrade and retrograde tracking technique, we successfully performed PCI for the RCA CTO under intravascular ultrasound (IVUS) guidance, implanting four drug-eluting stents (2.25 × 26 mm, 2.5 × 33 mm, 2.75 × 23 mm and 3.0 × 23 mm, *Figure 2 B11* and Moving image  *S15*). The patient was symptom-free and was discharged 3 days later. During the twelve-month follow-up period, the patient was free from myocardial ischemia symptoms and major adverse cardiovascular events (MACE).

## Discussion

The successful recanalisation of this complex RCA CTO hinged on two key elements: first, the comprehensive delineation of RCA collateral channels afforded by prior revascularisation of the LAD and LCX CTOs; and second, an exhaustive angiographic review undertaken at a moment of technical impasse.

Multi-vessel CTOs constitute a foremost challenge for complete interventional revascularisation. This is largely due to lesion anatomy that often fails to provide collateral channels suitable for procedural manipulation. The retrograde approach, whose technical success is contingent upon guidewire navigation through these collaterals,^8,9^ faces a critical limitation here. This critical limitation precludes its application as a standard strategy. Additionally, in CTOs where antegrade dissection re-entry (ADR) is anatomically precluded, conventional antegrade techniques—including antegrade wire escalation (AWE), the parallel wire technique, and IVUS-guided puncture—often struggle to achieve high success rates.

It is recognized that in acute coronary syndrome, culprit lesion PCI can expose collateral channels perfusing a CTO located in a non-culprit artery.^10^ An analogous mechanistic principle might be operative in multi-vessel CTOs. While collateral pathways in single-vessel CTO are relatively well-characterized,^3^ multi-vessel CTOs may result from a more profound ischemic preconditioning stimulus, thereby encouraging the development of complex, inter-vessel collateral circuits—including those traversing small vessels. Nevertheless, the *in vivo* manifestation of this collateral complexity is frequently masked by hemodynamic adaptations, including regional flow redistribution and perfusion pressure changes.

The therapeutic uncertainty surrounding coronary small-vessel disease, attributable to a lack of conclusive prognostic data, is further compounded in the setting of small-vessel CTOs. This case serves to highlight a distinct strategic value for PCI in small-vessel CTOs: the potential collateral channels exposed can provide potential retrograde access to facilitate revascularisation of subsequent, more complex CTOs. This finding should not be misconstrued as a recommendation for routine intervention on all small-vessel CTOs. Instead, it underscores the need for prudent judgment, weighing the not-insignificant procedural risk and complexity against the anticipated benefit, which may be strategic rather than solely geared towards symptomatic relief and improved prognosis. Identifying predictive factors to guide this nuanced decision-making process represents an important direction for future research.

This case also highlights that a comprehensive review of all angiograms before addressing residual CTO lesions can yield critical insights for procedural planning.

## Conclusion

Recanalising a small vessel CTO in multi-vessel disease unlocks the potential of its collateral channels, thereby paving the way for a retrograde approach to a more complex CTO.

## Lead author biography


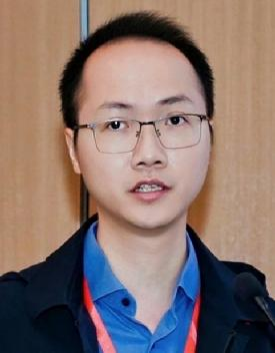


Zizhuo Su, MD, PhD, is an Interventional Cardiologist at Sun Yat-Sen Memorial Hospital, Sun Yat-Sen University in Guangzhou, China. He specializes in coronary intervention, with a particular expertise in the recanalisation of chronic total occlusions (CTO).

## Supplementary Material

ytag241_Supplementary_Data

## Data Availability

Data sharing is not applicable to this article because no datasets were generated or analysed during the preparation of this case report.

## Supplementary material

**Consent:** The authors confirm that written consent for submission and publication of this case report has been obtained from the patient, in line with COPE guidelines.
