# Supplementary material for: Rethinking small vessel CTOs: collateral channels as a bridge to subsequent complex intervention-a case report
Source: Eur Heart J Case Rep. 2026 Apr 21;10(4):ytag241. doi: 10.1093/ehjcr/ytag241 (PMC13096802; doi:10.1093/ehjcr/ytag241)
Supplement: ytag241_Supplementary_Data [file ytag241_supplementary_data.zip › Supplementary captions.docx]

**Moving image legends**

**Moving image 1 to 7：**Initial coronary angiography

**Moving image 8:** Angiogram of post-LAD PCI

**Moving image 9:** Angiogram of post-LCX PTCA

**Moving image 10 to 12:** Dual injection and collateral assessment for RCA CTO

**Moving image 13 and 14:** Retrograde guidewire Sion and microcatheter Caravel sequentially crossed collateral channel to the PLV

**Moving image 15:** Final angiogram of post-RCA PCI
